# Comparative Assessment of the Impacts of Wildland–Urban Interface Fire Ash on Growth of the Diatom *Thalassiosira weissflogii*

**DOI:** 10.3390/nano15060422

**Published:** 2025-03-09

**Authors:** Talal Alshehri, Amar Yasser Jassim, Bo Cai, Tammi L. Richardson, Mohammed Baalousha

**Affiliations:** 1 Center for Environmental Nanoscience and Risk, Department of Environmental Health Sciences, Arnold School of Public Health, University of South Carolina, Columbia, SC 29208, USA; talal@email.sc.edu (T.A.); amar.yasser@uobasrah.edu.iq (A.Y.J.); 2 Environmental Health Department, College of Public Health, Imam Abdulrahman Bin Faisal University, Dammam 31441, Saudi Arabia; 3 Department of Marine Vertebrates, Marine Science Center, University of Basrah, Basrah 61004, Iraq; 4 Department of Epidemiology and Biostatistics, Arnold School of Public Health, University of South Carolina, Columbia, SC 29208, USA; bocai@mailbox.sc.edu; 5 Department of Biological Sciences and School of the Earth, Ocean, and Environment, College of Arts and Sciences, University of South Carolina, Columbia, SC 29208, USA; richardson@biol.sc.edu

**Keywords:** wildland-urban interface fire, ash, nanoparticles, potentially toxic elements, diatom, environmental impact

## Abstract

Fires at the wildland–urban interface (WUI) result in the release of ash into the atmosphere that can be transported for long distances and deposited on land and in oceans. Wildfire ash has the potential to increase phytoplankton biomass in the open ocean by providing both major nutrients and trace metals. However, fires that originate at the WUI contain potentially toxic concentrations of metals such as Ti, Cr, Cu, Pb, and Zn, especially in coastal oceans close to WUI fires, where ash deposition rates are high. Here, we investigated the impact of fire ash from different sources originating from vegetation, structures, and vehicles on growth of the diatom *Thalassiosira weissflogii* (*T. weissflogii*). The diatom was exposed to ash suspensions containing equimolar concentrations of 10 and 50 µM Fe. The concentration of potentially toxic metals (e.g., Ti, Cu, and Zn) in the exposure suspensions decreased following the order vehicle ash suspension > structural ash suspension > vegetation ash suspension. Growth rates (GR) of *T. weissflogii* were between 0.44 d^−1^ and 0.52 d^−1^ in the controls, and varied with ash types, following the order vegetation (GR = 0.40 d^−1^ to 0.48 d^−1^) > vehicle (GR = 0.06 d^−1^ to 0.46 d^−1^) > structure (GR = 0.02 d^−1^ to 0.31 d^−1^) ash. Two ash samples (A 131 and A136) completely inhibited the growth of *T. weissflogii*, possibly due to high Ti, Cu, and Zn concentrations in the form of (nano)particles. Overall, this study showed that structural and vehicle ash, with high concentrations of potentially toxic metals, significantly suppress the growth of *T. weissflogii*, whereas vegetation ash with high concentrations of Fe and Mn but low concentrations of potentially toxic metals had no significant beneficial or suppressive effect. High concentrations of the metals Ti, Cu, and Zn in the form of nano(particles) in structural and vehicle ash are possible sources of toxicity to diatom growth. This study provides valuable insights into the potential impacts of WUI fires on aquatic ecosystems and can inform management strategies aimed at reducing these impacts.

## 1. Introduction

Wildfire is a natural process that has occurred in many ecosystems worldwide since the evolution of land plants and the increase in atmospheric oxygen level some 250 to 400 million years ago [1]. Recently, wildfires have increased in frequency, size, and severity due to climate change, preponderance of fuel, and the spread of fires into the built environment [2]. The latter are known as fires in the wildland–urban interface (WUI). Concerns about the destructive effects of WUI fires have increased in the United States as burned areas have increased from 5260 km^2^ in 1983 to 28,732 km^2^ in 2021, with the 2020 fire season burning a record 40,873 km^2^ of wilderness and damaging almost 18,000 structures, including 9600 residences [3].

Fire at the WUI transforms fuels (i.e., vegetation, soil organic matter, and construction material) into materials with different chemical and physical properties, including carbon dioxide and ash [4]. Ash is the particulate matter lofted and transported by air or deposited on the ground; it consists of mineral and charred organic materials [4]. In the wild, ash is derived dominantly from the combustion of vegetation and soil organic matter. In the urban environment, ash is derived from a wide variety of sources, including building materials (wood, roofing, vinyl, plumbing, electrical, paints, and solder materials), household items (furniture, cooking utensils, ceramics, rubber, and plastics), industrial solvents, petroleum products, automotive components, tires, and consumer electronics. Combustion of biomass and building materials at the WUI releases a complex mix of particles, liquids, and gaseous compounds. Contaminants released by WUI fires include pyrogenic carbon, polycyclic aromatic hydrocarbons (PAHs), polychlorinated dioxins and furans (PCDD/FE), volatile organic compounds (VOCs), metals, incidental nanomaterials (unintentionally formed nanomaterials as a result of direct or indirect human influence), environmentally persistent free radicals (EPFRs), nutrients, and other contaminants [5,6,7,8]. Thus, WUI fire ash tends to have higher concentrations of contaminants, in particular metal-bearing (nano)particles (e.g., Al, As, Cr, Cu, Fe, Ti, Zn, etc.) compared with vegetation ash [9,10].

Wildland–urban interface fires can eject enormous amounts of ash into the atmosphere which can be deposited on land and in oceans. The importance of WUI fire ash as a source of nutrients and potentially toxic metals in the ocean, however, has been largely overlooked compared to the much better studied effects of desert dust inputs [11]. This is mainly because of the unpredictability of WUI fires, and the difficulties associated with field sampling during and immediately after fires. Therefore, direct measurements of the effect of WUI fire ash on marine phytoplankton in the field are very scarce [12,13]. Also, there is growing evidence from satellite data that phytoplankton productivity in the open ocean increases after wildfires [14,15]. A main factor suggested for wildfire ash deposition-induced phytoplankton productivity is ocean fertilization with iron [14,15], the trace metal that limits phytoplankton productivity in large regions of the world’s oceans [16]. Algal blooms, such as those by filamentous algae (*Cladophora*), have also been observed after wildfires near fresh waters [17,18,19,20]. However, other studies have reported either no changes or decreases in algal growth following wildfires [17,21,22,23,24,25,26,27,28,29]. Inhibition of algal growth has been attributed to increases in total organic compounds (TOC), sulfur dioxides (SO_2_), calcium, potassium, sodium, and cadmium, and turbidity [20,29,30,31,32,33,34]. Despite the presence of PAHs in wildfire ash, several studies demonstrated the limited contribution of PAHs to wildfire ash toxicity, which was attributed to PAHs’ low solubility in water and thus low bioavailability [35,36]. While the referenced studies investigated the impact of ash from wildfires on aquatic organisms [20,26,29,30,31,32,33,34,37,38], none has investigated the impact of ash generated from urban materials, including structural and vehicle sources.

Besides iron, WUI fire ash releases the macronutrients NH_4_^+^, NO_3_^−^, PO_4_^3−^, and SiO_2_; volatile compounds such as Br^−^, Cl^−^, F^−^, and SO_4_^2−^; and a variety of trace metals such as Al, Cr, Cu, Mn, Ni, Pb, and Zn [9,10,39]. Some of these trace metals, especially Cd, Cu, and Zn, have potentially toxic effects at elevated concentrations [40]. One study showed toxic effects on *Thalassiosira weissflogii* (*T. weissflogii*) following the order Cd > Cu > Zn based on cellular metal concentrations [40]. Copper and Zn have been shown to decrease chlorophyll-*a* content in *T. weissflogii*, presumably because these metals inhibit the reductive steps in the biosynthesis pathway of photosynthetic pigments [40]. Increases in Cu (both ionic and nanoparticulate forms) concentrations have been shown to inhibit the growth of *T. weissflogii*, decrease the efficiency of its photosynthetic apparatus, change its cell shape and granularity, disturb its membrane integrity, increase its cell volume, increase the abundance of dead/inactive cells in the culture, enlarge and deform its nuclei, increase reactive oxygen species production, suppress enzymatic activity, and increase the depolarization of the mitochondrial membrane [41,42].

This study aimed to quantify the impacts of WUI residual (remaining on the ground) fire ash from different sources (vegetation, structure, and vehicles) and therefore different metal compositions and concentrations on the growth of the diatom *T. weissflogii* as a representative species [43]. *T. weissflogii* is ubiquitous and abundant in the Atlantic and Pacific oceans and in the Baltic Sea. Its responses to WUI fire ash, therefore, should be similar to those of natural populations of diatoms found in these regions. We hypothesized that while Fe has been known to stimulate ocean productivity, combinations of metals—especially potentially toxic metals from structural and vehicle ashes—may have non-stimulatory effects on diatom growth. Primary productivity by marine diatoms forms the foundation of coastal ocean food webs [44], and the availability of trace metals like Fe often limits this productivity [45]. Thus, inputs of metals from WUI are of great interest to ecologists and biological oceanographers.

## 2. Methods and Materials

### 2.1. Ash Collection, Analysis, and Stock Suspensions

Nine residual WUI fire ash samples were collected from October to November in 2020 following the Sonoma-Lake-Napa Unit (LNU) Lighting Complex Fire, which burned 1470 km^2^ and 1491 structures between 17 August and 2 October 2020. A detailed description of the LNU lightning complex fire is given elsewhere [9]. Residual ash was collected from burned vegetation (A31, A51, and A81), structures (A91, A124, and 131), and vehicles (A13, A134, and A136, Table 1) to assess the environmental impacts of these distinct sources.

Vegetation ash samples were collected from burned vegetation in forested areas far away from any human-made structures, eliminating the potential contribution of structural and vehicle ashes to the vegetation ashes. Structural ash samples were collected from specific locations within burned residential structures and vehicle ash samples were collected from inside or underneath burned vehicles, minimizing the potential contribution of vegetation ashes to the collected structural and vehicle ashes. Residual ash remaining on the ground was used in this study instead of fly ash because it was impossible to collect fly ash from specific sources due to safety concerns. Collecting fly ash from a specific source requires sampling at close proximity to the source due to mixing of fly ash from different sources in the atmosphere. Such sampling is not allowed during active fires.

All ash samples were collected prior to any rain or other precipitation, stored in the dark, and were shipped in a cooler to the University of South Carolina for further analysis. All ash samples were homogenized using a mortar and a pestle, then sieved using a 10-mesh 2 mm pore size nylon sieve (Zhangxing Instrument, Hangzhou, Zhejiang, China) to remove large particles. Homogenizing and sieving were carried out to minimize variability among replicate measurements of metal concentrations and *T. weissflogii* growth and to simulate a worst-case scenario regarding the release of contaminants from ash to marine water. Homogenized and sieved samples were stored in 15 mL acid-washed centrifuge tubes in a −20 °C freezer, then were digested using multi-wave microwave digestion (Anton Paar, Graz, Australia), and analyzed for total metal (Ti, V, Cr, Mn, Fe, Co, Ni, Cu, Zn, As, Se, Mo, Ag, Cd, Sn, Sb, and Pb) concentrations using an inductively coupled plasma-time of the flight-mass spectrometer (ICP-TOF-MS from TOF-WERK, Switzerland) as described elsewhere [9] and summarized in the supplementary information. Ash stock suspensions were prepared by suspending different masses (50 to 300 mg) of ash in 20 mL ultrapure water (Table S3) to obtain WUI fire ashes with 10 and 50 µM Fe equimolar concentrations. This selection of ash content based on iron concentration was made to test the study hypothesis and to cover the range of potential ash and iron concentrations in the ocean as a result of ash deposition. For instance, a previous study estimated that if all ashes generated from the Marchal fire (burned area of 24.4 km^2^) were deposited in the Santa Barbara channel (approximately 100 km × 40 km) it would result in ash concentration of 0.01–0.03 g L^−1^ in the top 20 m of the Santa Barbara channel [46]. In this study, we used a broader ash concentration range (e.g., 0.0085 to 0.12 and 0.04 to 0.6 g L^−1^ for the 10 and 50 equimolar Fe concentrations) to cover the potential variability in ash and thus Fe concentrations in the ocean. Additionally, the Marchal fire was a very small fire compared to LNU Lightning Complex Fire, A = 1470 km^2^), which could result in higher ash deposition in the ocean. Therefore, our treatments were probably similar to conditions experienced during wildfires. The ash suspensions were mixed for 24 h at 60 rpm using a tube rotator (Fisherbrand^TM^, Fisher Scientific, Pittsburgh, PA, USA) followed by 30 min sonication using a bath sonicator (Branson 2800, 40 kHz, Danbury, CT, USA). The ash suspensions were not filtered. The pH of the stock suspension of all ashes varied within a very narrow range of 8.02 to 8.12 with a mean of 8.07 ± 0.03.

### 2.2. Algal Culture

Cultures of *T. weissflogii* (CCMP 1051) were obtained from the National Center for Marine Algae and Microbiota at the Bigelow Laboratory for Ocean Sciences (East Boothbay, ME, USA). Stock cultures were grown in f/2 medium [47,48] prepared using 0.2 µm-filtered oligotrophic ocean seawater collected from the Bermuda Atlantic time-series station (located approximately 75 km southeast of Bermuda (at 31°50′ N, 64°10′ W), which was naturally low in nutrients and trace metals. Iron concentration in the seawater was confirmed by ICP-TOF-MS to be below the instrument detection limit (0.51 μg L^−1^). Cultures were grown in a reach-in Percival incubator at 20 °C on a 12 h:12 h light:dark cycle at ~150 µmoles photons m^−2^ s^−1^ photosynthetically available radiation (PAR) as measured with a Biospherical Instruments QS 2101 light meter (Biospherical Instruments, Inc., San Diego, CA, USA) just outside culture containers. All cultures were grown in 250 mL Pyrex flasks with no more than 150 mL of culture in each and were gently swirled by hand each day.

Experimental cultures were grown in f/2 as described above but with the trace metal solution omitted. All macronutrients (nitrate, phosphate, and silicate) and vitamins were present in normal f/2 concentrations.

### 2.3. Growth Experiment

Each of the nine ash samples was added to independent replicates (n = 8) of *T. weissflogii* culture. Each treatment included 10 and 50 μM Fe (equimolar concentration) and a control group without ash (Table S3). This selection of ash content based on iron concentration was made to test the hypothesis that while Fe is known to stimulate ocean productivity, combinations of metals—especially potentially toxic metals from structural and vehicle ashes—may have inhibitory effects on diatom growth. Algal growth experiments were performed in 250 mL Erlenmeyer flasks (Fisher, Waltham, MA, USA). Ten milliliters of *T. weissflogii* stock culture in exponential growth phase was added to approximately 90 mL of ocean water, followed by the addition of a given volume of ash stock suspension (Table S3) to achieve iron concentrations of 10 and 50 µM (referred to as 10 and 50 µM Fe treatments). After the addition of the ash suspension to the test medium, the flasks were gently stirred. Each experiment lasted for 96 h. All experiments were performed at room temperature (20 °C) on a 12 h:12 h light:dark cycle under ~150 µmoles photons m^−2^ s^−1^ PAR. Five milliliter samples were taken daily and chlorophyll fluorescence (excitation at 436 nm, emission at 670 nm) was measured using a Turner Designs 10-AU fluorometer (Turner Designs, Sunnyvale, CA, USA). Chlorophyll fluorescence is a reliable indicator of diatom growth because the amount of fluorescence light emitted by chlorophyll molecules is directly proportional to the concentration of the diatom present [49]. Thus, chlorophyll fluorescence is often used as a proxy for phytoplankton biomass and is a rapid and simple technique for determining phytoplankton growth [50]. As fluorescence per chlorophyll may vary with time of day, care was taken to sample cultures at the same time of day each day [51]. Culture growth rates (d^−1^) were calculated from the least-squares regression of fluorescence intensity versus time during exponential growth. For statistical analysis, a Kruskal–Wallis non-parametric ANOVA was used to determine whether growth rates were significantly different between controls and treatments. Differences were considered significant at *p* < 0.05. The Pearson test was used to determine whether the *T. weissflogii* growth rate exhibited linear correlation with metal concentration. The Spearman test was used determine whether metals without significant linear correlation with the *T. weissflogii* growth rate could have a nonlinear association with growth rate.

## 3. Results and Discussion

### 3.1. Physical and Chemical Characterization of WUI Fire Ash

**Figure 1:**
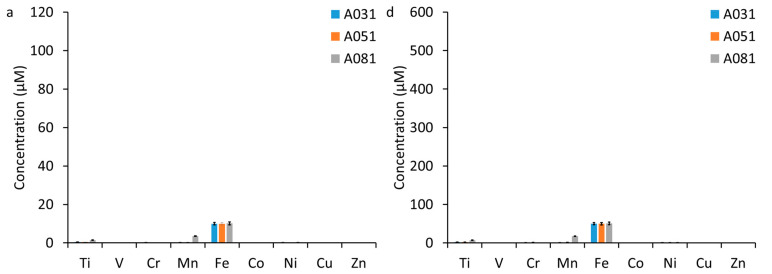

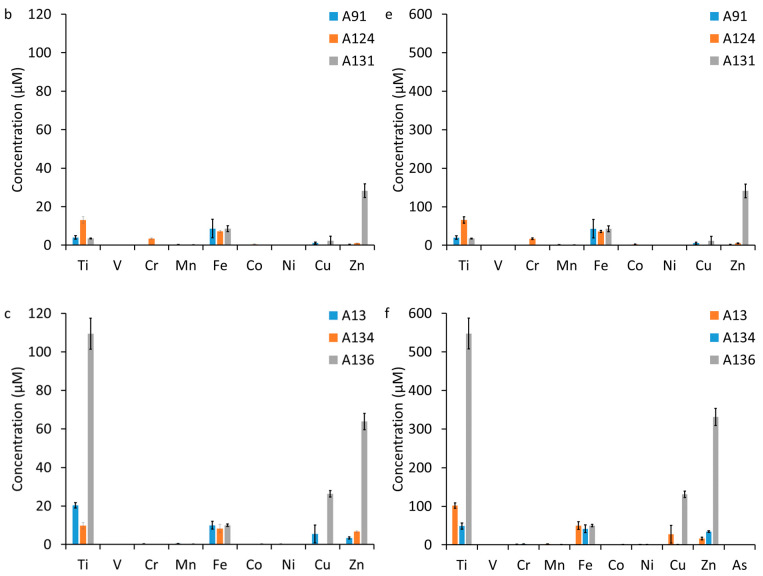
Concentrations of select metals in the test medium as a result of addition of fire ash from different sources (**a**,**d**) vegetation, (**b**,**e**) structures, (**c**,**f**) vehicles. The Fe content was fixed for all exposures at (**a**–**c**) 10 µM Fe equimolar and (**d**–**f**) 50 µM Fe equimolar. The numerical values of the concentrations of these metals along with As, Se, Mo, Ag, Cd, Sn, Sb, and Pb are also presented in Tables S4 and S5.

The total concentration of the most abundant elements in the test medium as a result of addition of WUI fire ash from different sources varied among the three ash sources (Figure 1 and Tables S4 and S5). In general, metal concentrations (e.g., Ti, Cu, Zn, and Pb) decreased following the order: test medium containing vehicle ash ≥ test medium containing structural ash > test medium containing vegetation ash. The trend of metal concentrations in the test medium suspensions was consistent with the trend of metal concentrations in the WUI fire ashes; that is, metal concentrations (e.g., Ti, Cu, Zn, and Pb) decreased following the order vehicle ash > structural ash > vegetation ash [9]. This trend in metal concentration is attributed to the source of the metals in the raw fuel [9]. Metals in vegetation are taken up by plants from soils with essential elements such as Fe and Mn occurring at highest concentrations in vegetation [52]. In contrast, in structures and vehicles, metals originate from their use in vehicle and structural materials. The high concentrations of Ti in vehicle and structural ashes are attributed to TiO_2_ pigments, the most widely used pigments in paints in vehicles and structures [53]. The high concentration of Zn in vehicle and structural ashes are attributed to their use as a vulcanizing agent in tire rubber production (e.g., Zn accounts for 1–2% of tire weight), and as a galvanizing agent to protect iron and steel from rusting in vehicles and structural material, and as white paint pigments [54]. Copper is widely used in vehicle parts, water pipes, and electrical wires [55,56]. The high concentration of Pb in vehicle and structural ash is attributed to their use in batteries and in old paint as a pigment [57]. In the vegetation ash containing test medium, Fe was the most concentrated element followed by Mn, Ti, Cr, and Ni (Figure 1a,d), with the highest Mn and Ti concentrations in A81. The high concentration of Fe compared to other metals in vegetation ash is consistent with Fe being an essential element for plants which has been reported to occur at high concentrations in forest fire ash [58,59]. In the structural ash-containing medium, Fe was the element highest in concentration followed by Ti, Zn, Cr, and Cu, with higher Ti concentrations in A124 and higher Zn in A131 (Figure 1b,e). In the vehicle ash-containing medium, Ti was the element highest in concentration followed by Zn, Fe, and Cu, with the highest Ti, Cu, and Zn in A136 (Figure 1c,f). Detailed spectroscopy and microscopy analyses of the studied ashes demonstrated that metals occurred dominantly as (nano)particles [9]. The vegetation ash samples (A31 and A81) contained mainly C, Ca, and O-bearing (nano)particles, most likely in the form of calcium carbonates. In contrast, the structural (A124 and A131) and vehicle (A13) ash samples contained primarily Al-silicate, Ca-sulfate, and Cu-, Ti- and Zn-bearing (nano)particles. The core diameter of the Ti-, Zn-, and Cu- bearing particles ranged from 50 to 500 nm, 50 to 250 nm, and 10 to 50 nm, respectively. The core size of other Ca-carbonates, Al-silicate, Ca-sulfate type were not quantified.

### 3.2. Diatom Growth

**Figure 2:**
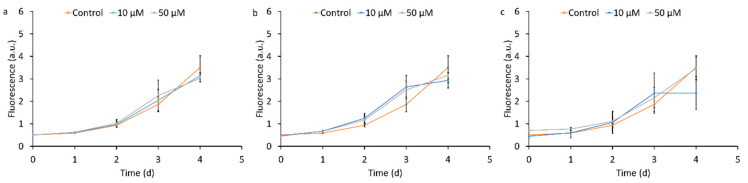

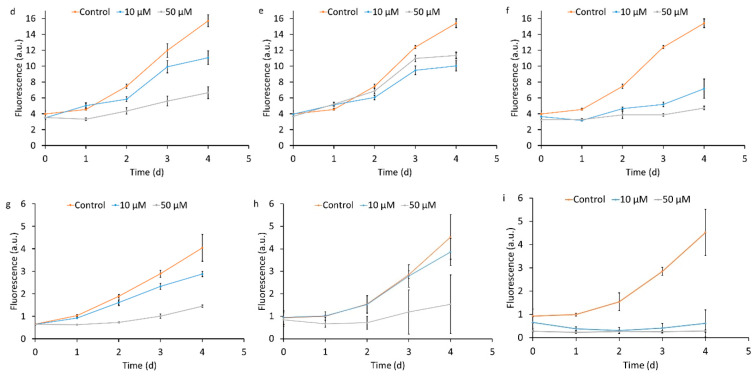
Growth curves of the *Thalassiosira weissflogii* following exposure to wildland–urban interface fire ash: (**a**–**c**) vegetation ash (**a**) A31, (**b**) A51, and (**c**) A81; (**d**–**f**) structural ash (**d**) A91, (**e**) A124, and (**f**) A131; and (**g**–**i**) vehicle ash (**g**) A13, (**h**) A134, and (**i**) A136. The concentration of Fe was maintained constant in all exposures at 10 µM and 50 µM. The errors are calculated as the standard deviation of 8 replicates. The experiments were performed on batches of three ash samples from the same source at a time.

Qualitatively, the addition of WUI fire ash altered the growth rates of *T. weissflogii* to different extents (Figure 2). The vegetation ash had no significant effects on *T. weissflogii* growth compared to the controls regardless of the ash (Fe) concentration (Figure 2a–c). In contrast, the structural and vehicle ash decreased the *T. weissflogii* growth compared to the controls (Figure 2d–i). Exposure to A91, A124, A13, and A134 reduced the growth of *T. weissflogii* at both ash concentrations (10 and 50 μM Fe treatments) relative to the control (Figure 2d,e,g,h). Exposure to A131 and A136 inhibited the *T. weissflogii* growth partially at 10 μM Fe treatments and fully at 10 μM Fe treatments (Figure 2f,i).

These findings are consistent with previous studies demonstrating the growth inhibition of wildfire runoff for a range of freshwater and marine organisms such as *Pseudokircheneriella subcapitata*, *Lemna minor*, and *Vibrio fischeri* [36,38]. Other studies demonstrated increased mortality of *Daphnia magna, Ceriodaphnia dubia*, *Danio rerio,* and *C. fluminea* due to exposure to wildfire ash, which was attributed to presence of unintended toxic substances in the ash including metals and PAHs [60,61] or to changes in pH and electrical conductivity [35]. Furthermore, fire ash has been shown to reduce the number of eggs per snail (the aquatic snail *Biomphalaria glabrata*), which was attributed to the high pH and possibly other unintended compounds present in the ash [62]. Other studies suggested that the inhibition of algal growth following wildfires was a result of increases in total organic compounds, sulfur dioxides (SO_2_), calcium, potassium, sodium, and cadmium, and turbidity [20,29,30,31,32,33,34]. These studies demonstrated that many compounds in the wildfire ash and runoff could affect the toxicity of fire ash and highlight the difficulty in pinpointing specific toxic agents in the fire ash.

**Figure 3:**
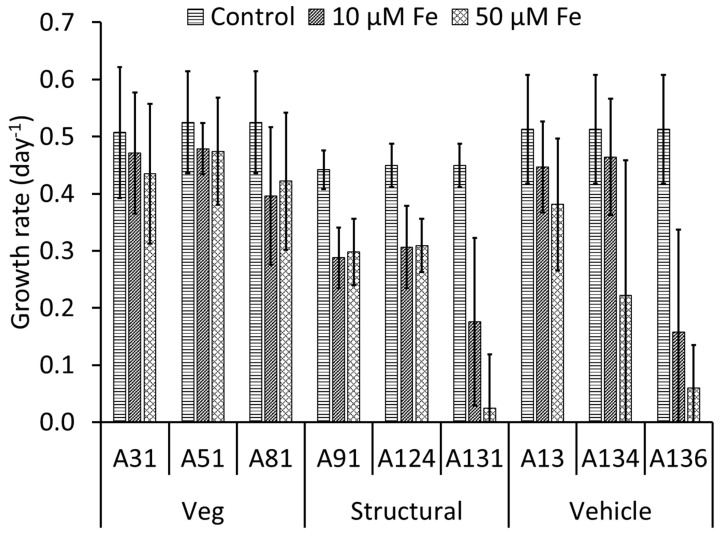
Growth rates of the diatom *Thalassiosira weissflogii* following exposure to fire ash. The error bars indicate the standard deviation of eight replicates.

Quantitatively, the *T. weissflogii* had a growth rate between 0.44 d^−1^ and 0.52 d^−1^ in the controls (Figure 3). The addition of the vegetation ash did not result in significant changes in the growth rate, which varied between 0.40 d^−1^ and 0.48 d^−1^, likely due to the low concentrations of potentially toxic metals (e.g., Zn and Cu, Figure 1a,d). In contrast, the addition of the structural ash resulted in a significant decrease in the *T. weissflogii* growth rate at 10 μM and 50 μM Fe treatments, which varied between 0.02 d^−1^ and 0.31 d^−1^. Similarly, the addition of vehicle ash resulted in a significant decrease in the *T. weissflogii* growth rate at 10 μM and 50 μM Fe treatments, which varied between 0.06 d^−1^ and 0.46 d^−1^. A134 did not result in a significant change in the *T. weissflogii* growth rate at 10 μM Fe treatment but resulted in a significant decrease in *T. weissflogii* growth rate at 50 μM Fe treatment. The decrease in *T. weissflogii* growth rate following the addition of structural and vehicle ash could be due to high concentrations of potentially toxic metals (e.g., Ti, Zn, and Cu, Figure 1b,c,e,f). Nonetheless, other ash constituents (such as major ions, organic chemicals, turbidity) that have not been measured in this study also might contribute to the reduction of *T. weissflogii* growth rate.

### 3.3. Fertilization vs. Toxic Effects of WUI Fire Ash: Role of Metals

Metals such as Cd, Co, Cu, Fe, Mn, Mo, Ni, and Zn are essential for phytoplankton growth as they are key components of pigments, enzymes, and other growth-regulating molecules [63,64]. Of these elements, Fe is often the element that phytoplankton requires faster than they are able to acquire it. Thus, Fe is the element that usually limits phytoplankton growth in remote regions of the ocean [65] and in some coastal regions [45] that do not receive Fe input from terrestrial sources or atmospheric deposition. Thus, the addition of Fe by WUI fire ash could enhance phytoplankton growth in coastal regions. Other trace metal nutrients (e.g., Co, Cu, Mn, and Ni) are required in lower concentrations and are thus supplied at rates that satisfy the biological demand. In addition to their nutritional roles, trace metals (e.g., Cu and Zn) have been shown to inhibit the growth of *T. weissflogii* and decrease chlorophyll-*a* content in *T. weissflogii* at high concentrations, presumably because these metals inhibit the reductive steps in the biosynthesis pathway of photosynthetic pigments [40].

In our study, the addition of vegetation ash did not enhance or inhibit the growth rate of in *T. weissflogii* compared to the controls (Figure 2a–c). This indicates that the vegetation ash either did not supply this species with sufficient micronutrients or that the concentration of toxic metals was high enough to depress any Fe-induced growth enhancement of this species. Given the low concentration of potentially toxic metals (e.g., Ti, Cu, and Zn) in the vegetation ash, which indicates limited contribution of these metals to the ash toxicity, we conclude that Fe did not enhance growth of *T. weissflogii*. In contrast to vegetation ash, structural and vehicle ash inhibited the growth of *T. weissflogii* compared to the controls, suggesting that the concentration of toxic metals such as Ti, Cu, and Zn in these ashes (Figure 1b,c,e,f) was high enough to depress the growth of this species [42,66]. Furthermore, vehicle and structural ash also might contain other constituents that have not been measured in this study, which may contribute to the reduction of the *T. weissflogii* growth rate.

Most structural and vehicle ash samples caused partial growth inhibition of *T. weissflogii*. Only two ash samples (A131 and A136) induced full inhibition of *T. weissflogii* at 50 μM Fe treatment. Additionally, exposure to 10 μM Fe treatment of A131 and A136 reduced *T. weissflogii* growth rate (0.18 and 0.16 d^−1^) significantly compared to the controls. These two ash samples were characterized by high Ti, Fe, Cu, and Zn, all occurring at several μM to 10s of μM in concentration. A131 and A136 (at 50 μM Fe treatment) were characterized by high Ti (17.4 and 547.4 μM), Cu (e.g., 11.3 and 131.5 μM), and Zn (e.g., 141.2 and 319.2 μM) concentrations close to or exceeding the median effect/inhibition concentration (EC50) of Ti (e.g., TiO_2_ nanoparticles extracted from sunscreen products (e.g., 12–62 μM Ti) for the marine diatom *Thalassiosira pseudonana* [67], and EC50 of Cu (e.g., 0.8 μM Cu^2+^ and 15.7 μM Cu in the form of CuO nanoparticles) [42] and Zn (e.g., 75 μM Zn^2+^ and 75 to 150 μM Zn in the form of ZnO nanoparticles) [66] for *T. weissflogii*. The other ash exposure media contained Cu and Zn concentrations lower than those that caused EC50 (Table S5). The much higher concentrations of Cu and Zn in the fire ash compared to the toxicity of the Cu^2+^ and Zn^2+^ and the lower toxicity of CuO and ZnO nanoparticles compared to the toxicity of Cu^2+^ and Zn^2+^ suggest that the toxicity of Cu and Zn in the fire ash can be attributed to their presence in fire ash as (nano)particles. This is consistent with the occurrence of Cu and Zn dominantly in the (nano)particulate form [9,10] in the fire ash. In the 50 μM Fe treatment, the test medium for some ash samples (e.g., A13, A124, and 134) contained high Ti concentrations (102.1, 65.4, and 48.7 μM Ti, respectively), which were very close or exceeding the EC50 of the TiO_2_ nanoparticles used in sunscreen. However, *T. weissflogii* did not display complete inhibition in these test media. Other forms of TiO_2_ particles, such as commercially available TiO_2_ manpower (<25 nm) and TiO_2_ pigments (100 to 300 nm) extracted from tooth paste, induced lower inhibition (e.g., 5–30% inhibition) of *Thalassiosira pseudonana* compared to TiO_2_ extracted from sunscreens [67]. The lower toxicity of TiO_2_ pigments is consistent with the pigment size of TiO_2_ particles observed in the WUI fire ash. The silica frustule of *T. weissflogii* have been shown to be susceptible to exposure to ZnSO_4_ because excess uptake of Zn^2+^ competes for uptake of silica by algal cells and consequently impairs formation of frustules and growth [68].

Linear and non-linear correlation tests were performed to further examine the relationships between *T. weissflogii* growth rate and metal concentrations. The Pearson’s test demonstrated that the *T. weissflogii* growth rate exhibited significant negative linear associations with the concentration of Ti, V, Cu, Zn, As, Se, Mo, Ag, Cd, and Sb (Table S6). In contrast, the *T. weissflogii* growth rate did not exhibit a significant linear correlation with the concentration of Cr, Mn, Fe, Co, Ni, Sn, or Pb. Furthermore, for those metals without significant linear correlation with growth rate, the Spearman’s test demonstrated that *T. weissflogii* growth rate did not exhibit a significant non-linear correlation with the concentration of Cr, Mn, Fe, Co, or Ni (Table S6). In contrast, the *T. weissflogii* growth rate exhibited a significant non-linear negative correlation with the concentrations of Sn and Pb.

Furthermore, despite the seemingly important role of metals in determining the toxicity of the WUI fire ash studied here, other chemicals in the WUI fire ash that were not measured in this study could play an important role. However, screening for the multitude of chemicals that can be present in WUI fire ashes is beyond the scope of this study.

## 4. Conclusions and Future Perspective

This study investigated the impact of fire ash from different sources originating from vegetation, structures, and vehicles on growth of the diatom *Thalassiosira weissflogii*. The diatom was exposed to ash suspensions containing 10 and 50 µM Fe (equimolar Fe concentrations). Metal (e.g., Ti, Cu, and Zn) concentrations decreased following the order vehicle ash suspension > structure ash suspension > vegetation ash suspension. Vegetation ash, dominantly rich in Fe and Mn-bearing (nano)particles with low concentrations of potentially toxic metals, did not significantly enhance or suppress *T. weissflogii* growth rates. In contrast, structural and vehicle ash, rich in potentially toxic metals such as Ti, Cu, and Zn-bearing (nano)particles, significantly reduced *T. weissflogii* growth, thus supporting our overall hypothesis. These findings highlight the potential importance of metal nano(particles) in determining fire ash toxicity and thus highlight the need for future research to develop in-depth mechanistic understanding of how metal-bearing nano(particles) modulate fire ash toxicity. This is particularly important because metals in fire ash exist in different forms (e.g., nanoparticles, complexed by various ligands) and oxidation states (e.g., oxidized, reduced, or mixed oxidized/reduced species) [39,69,70], which are heavily impacted by fuel source and fire conditions, affecting their bioavailability and toxicity [71]. Thus, additional studies are needed to decipher the impact of these parameters (e.g., fuel source, fire conditions, and metal transformations during combustion), the effect of mixture of metals nano(particles) on marine organisms as well as the impact of other contaminants (e.g., polycyclic aromatic hydrocarbons (PAHs), polychlorinated dioxins and furans (PCDD/FE), environmentally persistent free radicals (EPFRs) [5,6,7,8,72]) in the fire ash that have not been considered in this study. While we focused on a representative marine diatom for this study, it is possible that growth responses of different phytoplankton groups (e.g., dinoflagellates, haptophytes, cyanobacteria) are more sensitive or responsive [46]. If ash deposition results in changes in phytoplankton community composition, this could substantially influence food web dynamics and fisheries in coastal oceans [73]. Further work is needed to address the large spatial scale or long-time scale effects of atmospheric deposition of wildfire ash.

## Figures and Tables

**Table 1:** Description of the ash samples collected following LUN Lightning Complex Fire that burned during the 2020 California fire season.

Sample Number	Sampling Date	Ash Source	Sample Description
A31	8 October 2020	Vegetation	Burned chaparral (charred manzanita and some pine) site on gentle slopes; serpentinite unit
A51	8 October 2020	Vegetation	Burned chapparal (manzanita), white ash with some black ash; sandstone unit
A81	8 October 2020	Vegetation	Burned oak, white ash with some black ash; bedrock undetermined
A91	15 October 2020	Structure/residential	Hot tub/spa
A124	15 October 2020	Structure/residential/paint	Shed—house paint, some wires
A131	16 October 2020	Structure	Burned barn—redwood, galvanized steel, tires, Cu wire
A13	7 October 2020	Vehicle	Burned trailer—south end
A134	16 October 2020	Vehicle	Burned boat and trailer—fiberglass, aluminum, tires
A136	16 October 2020	Vehicle	Burned trailer (for 2 horses)—tires, Cu wire, aluminum, book (manual)

## Data Availability

Data will be made available on request.

## Supplementary Materials

The following supporting information can be downloaded at: https://www.mdpi.com/article/10.3390/nano15060422/s1. Table S1: TOFWERK ICP-TOF-MS operating conditions; Table S2: Accuracy of the analytical results: Mean and standard deviation of three replicate analysis of standard reference ash materials (NIST SRM 1663C, and BCR-176R) in comparison to certified reference, reference, and informational values provided in the certificate of analysis; Table S3: Preparation of ash stock suspension (20 mL ultrapure water) and exposure medium (100 mL Bahamas seawater); Table S4: Concentrations of metals (µM) in the test medium as a result of addition of fire ash from different sources including vegetation, (A31, A51, and A81) structures, (A91, A124, and A131) vehicles (A13, A134, and A136). The Fe content was fixed for all exposures at 10 µM Fe; Table S5: Concentrations of metals in the test medium as a result of addition of fire ash from different sources including vegetation, (A31, A51, and A81) structures, (A91, A124, and A131) vehicles (A13, A134, and A136). The Fe content was fixed for all exposures at 50 µM Fe; Table S6: Pearson and spearman correlation analysis between metal concentrations and *Thalassiosira weissflogii* growth rate (GR).
