# Supplementary material for: Comparative Assessment of the Impacts of Wildland–Urban Interface Fire Ash on Growth of the Diatom Thalassiosira weissflogii
Source: Nanomaterials (Basel). 2025 Mar 9;15(6):422. doi: 10.3390/nano15060422 (PMC11944898; doi:10.3390/nano15060422)
Supplement: Supplementary file 1 [file nanomaterials-15-00422-s001.zip › nanomaterials-3478570-supplementary.pdf]

Supporting Information for

Comparative assessment of the impact of wildland-urban interface fire ash on growth of the diatom *Thalassiosira weissflogii*

Talal Al Shehri[1, 2], Amar Yasser Jassim[1,3], Bo Cai[4], Tammi Richardson[5], and Mohammed Baalousha[1*]

[1]Center for Environmental Nanoscience and Risk, Department of Environmental Health Sciences, Arnold School of Public Health, University of South Carolina, Columbia, South Carolina, United States

[2]Environmental Health Department, College of Public Health, Imam Abdulrahman Bin Faisal University, Dammam 31441, Saudi Arabia

[3]Department of Marine Vertebrates, Marine Science Center, University of Basrah, Iraq

[4]Department of Epidemiology and Biostatistics, Arnold School of Public Health, University of South Carolina, Columbia, South Carolina, United States

[5]Biological Sciences Department, College of Arts and Sciences, University of South Carolina, Columbia, South Carolina, United States

* Corresponding author: mbaalous@mailbox.sc.edu

Digestion and Metal Analysis

One hundred mg of each ash sample was weighed into a polytetrafluoroethylene (PTFE) digestion vessel. Digestion reagents were added in the following order: 9 ml of distilled HNO_3, 3 ml of distilled HF, and 2 ml of H_2O_2. The acid digestion was performed in a Multiwave microwave (Multiwave Pro, Anton Paar, Graz, Austria) at a constant power of 1500 W for 60 minutes, preceded by a 15-minute ramping time to reach the desired power. The digestate was then evaporated in two steps using the same microwave system to remove non-reacted HF. The first evaporation was performed with 10 minutes of ramping time followed by 9 minutes of holding time at 1500 W. Then, 3 ml of distilled HNO_3 was added into the vessel to dissolve any insoluble fluoride salts. The second evaporation was performed with 10 minutes of ramping time followed by 3 minutes of holding time at 1500 W. The digested samples were then diluted in 10% HNO_3 (trace metal grade, Fisher Chemical, Fair Lawn, NJ, USA) and stored until total metal analysis.

Total metal concentrations were determined using an inductively coupled plasma-time of flight-mass spectrometer (ICP-TOF-MS, TOFWERK, Switzerland). Mass spectra calibration and routine tuning were performed prior to analysis every day to achieve maximum sensitivity. Elemental concentration calibration was established using a series of ionic standards prepared in 1% HNO_3 from commercially available ICP multi-element standards (BDH Chemicals, Radnor, PA, USA). Internal standards (ICP Internal Element Group Calibration Standard, BDH Chemicals, Radnor, PA, USA) were applied to monitor signal drift for quality control. The instrument operating conditions are presented in **Table S2,** and the monitored isotopes are listed in **Table S3**. Dissolved multi-element standards were prepared in 1% HNO_3 from commercially available ICP standards (BDH Chemicals, Radnor, PA, USA), with concentrations ranging from 0.001 to 100 μg L^{-1}. Internal standards (ICP Internal Element Group Calibration Standard, BDH Chemicals, Radnor, PA, USA) were monitored simultaneously for quality control. All isotopes were analyzed in collision mode with a helium and hydrogen gas mixture.

The recovery of the digestion procedure was determined by digesting and analyzing two standard reference materials for trace elements in coal fly ash: NIST SRM 1663C (NIST, Gaithersburg, MD, USA) and BCR-176R (IRMM, Retieseweg, Geel, Belgium). For 1663C, the recovery varied between 82% and 137% (**Table S3**). For BCR-176R, the recovery varied between 77 and 131%. All elements' relative standard deviation was <15%, indicating good precision.

Table S1. TOFWERK ICP-TOF-MS operating conditions.

Instrument parameter	Value				
Plasma Power	1550 V				
Nebulizer Gas Flow	1.1 L/min				
Auxiliary Gas Flow	0.8 L/min				
Cooling Gas Flow	14 L/min				
Injector Diameter	2.5 mm				
Collision Cell Gas	5 mL/min He with 4.5% H_2				
CCT Bias	-2.50 V				
Notch	Mass	29	32	36.3	41

	Amplitude (V)	1.6	2.0	2.0	1.2
TOF Repetition Rate	33 kHz				
Detected Mass Range	14-275 m/Z				
(CeO/Ce)	< 3.0%				
Data Acquisition	Continuous Mode				
TOF Time Resolution	0.3 s				

Table S2. Accuracy of the analytical results: Mean and standard deviation of three replicate analysis of standard reference ash materials (NIST SRM 1663C, and BCR-176R) in comparison to certified reference, reference, and informational values provided in the certificate of analysis.

		Standard concentrations (mg kg^{-1})		Measured concentrations (μg kg^{-1})				Recovery (%)			
				Mean		Standard deviation		Mean		Standard deviation	
**Element**	**Isotope**	**1663C**	**176R**	**1663C**	**176R**	**1663C**	**176R**	**1663C**	**176R**	**1663C**	**176R**
Ti	49Ti	1663C	176R	1663C	176R	1663C	176R	113		0.7	
V	51V	7240.00		8191.55	14629.31	53.13	720.83	92	96	0.4	4.8
Cr	52Cr	286.20	35.00	263.35	33.66	1.27	1.67	83	94	0.7	2.7
Mn	55Mn	258.00	810.00	213.47	759.02	1.74	21.50		103		6.3
Fe	56Fe	240.20	730.00		752.18		46.02	115	122	1.3	2.9
Co	59Co	104900.00	13100.00	120721.61	15958.33	1311.88	376.47	125	102	0.5	4.4
Ni	60Ni	42.90	26.70	53.75	27.12	0.19	1.17	120	92	0.8	2.9
Cu	65Cu	132.00	117.00	158.17	107.37	1.01	3.40	137	100	1.4	4.7
Zn	66Zn	173.70	1050.00	237.91	1052.34	0.00	48.96		131		6.0
As	75As	235.00	16800.00		21994.64		1009.76	100	100	0.9	4.6
Se	78Se	186.20	54.00	186.14	54.03	1.66	2.48		146		6.2
Mo	98Mo			23.33	11.95	0.06	0.52				
Ag	107Ag			11.22	31.04	0.19	1.54		94		3.0
Sn	120Sn	0.76	226.00	3.06	191.14	0.18	8.15				
Sb	121Sb			12.86	1231.79	0.22	51.22	104	96	3.3	4.3
Pb	208Pb		28.30	5.90	23.72	0.09	1.00	82	77	0.5	3.7

Standard values in black indicate certified reference values, in red indicate reference values, and in blue indicate informational values.

Table S3. Preparation of ash stock suspension (20 mL ultrapure water) and exposure medium (100 mL Bahamas seawater).

	**Stock suspension**				**Exposure medium**			
**Ash #**	**Ash mass (mg)**	**Ultrapure water volume (mL)**	**Ash concentration in stock suspension (g/L)**	**Fe concentration in stock suspension (mM)**	**Volume of ash stock suspension used to prepare 10 uM (mL)**	**Ash concentration g/L for 10 uM Fe**	**Volume of ash stock suspension used to prepare 50 uM (mL)**	**Ash concentration g/L for 50 uM Fe**
**A31**	50	20	2.5	2.9	0.34	0.01	1.702	0.04
**A51**	50	20	2.5	2.2	0.458	0.01	2.291	0.06
**A81**	300	20	15	1.4	0.727	0.11	3.637	0.55
**A91**	70	20	3.5	1.2	0.832	0.03	4.16	0.15
**A124**	70	20	3.5	3.2	0.313	0.01	1.565	0.05
**A131**	70	20	3.5	1.4	0.733	0.03	3.664	0.13
**A13**	60	20	3	1.3	0.768	0.02	3.84	0.12
**A134**	60	20	3	2	0.49	0.01	2.452	0.07
**A136**	300	20	15	1.2	0.802	0.12	4.011	0.60

Table S4. Concentrations of metals (μM) in the test medium as a result of addition of fire ash from different sources including vegetation, (A31, A51, and A81) structures, (A91, A124, and A131) vehicles (A13, A134, and A136). The Fe content was fixed for all exposures at 10 μM Fe.

**Mean**	**Ti**	**V**	**Cr**	**Mn**	**Fe**	**Co**	**Ni**	**Cu**	**Zn**	**As**	**Se**	**Mo**	**Ag**	**Cd**	**Sn**	**Sb**	**Pb**
A031	0.48	0.013	0.22	0.20	9.99	0.0121	0.1734	< DL	< DL	< DL	0.0008	< DL	< DL	< DL	0.0005	< DL	0.0005
A051	0.42	0.016	0.24	0.33	10.00	< DL	0.1818	< DL	0.0845	0.0020	0.0015	0.0000	0.0009	0.0006	0.0004	< DL	< DL
A081	1.45	0.024	< DL	3.53	10.16	< DL	0.1822	< DL	< DL	0.0048	0.0042	0.0003	< DL	< DL	0.0043	< DL	< DL
A91	4.00	0.017	0.05	0.24	8.57	0.0038	0.0238	1.1130	0.3407	0.0025	0.0016	0.0005	0.0011	0.0030	0.0387	0.0269	0.0318
A124	13.08	0.011	3.40	0.04	7.14	0.4953	0.0146	0.0201	1.0320	0.0021	0.0024	0.0009	0.0006	0.0011	0.0054	0.0040	0.0007
A131	3.48	0.024	0.10	0.12	8.57	< DL	0.0287	2.2567	28.2478	0.0084	0.0093	0.0013	0.0010	0.0084	0.0134	0.0231	0.1419
A13	20.42	0.015	0.17	0.49	10.00	0.0112	0.1515	5.4782	3.3864	< DL	0.0059	0.0011	0.0007	0.0011	0.0251	0.0466	1.9449
A134	9.73	0.007	< DL	0.07	8.33	0.0293	0.0878	0.1825	6.6691	< DL	0.0024	0.0027	< DL	0.0004	0.0077	0.0264	0.0109
A136	109.44	0.047	0.09	0.21	10.00	0.2227	0.0520	26.2913	63.8300	0.0176	0.0235	0.0031	0.0012	0.0025	0.0210	0.2794	0.0552
**Standard deviation**	**Ti**	**V**	**Cr**	**Mn**	**Fe**	**Co**	**Ni**	**Cu**	**Zn**	**As**	**Se**	**Mo**	**Ag**	**Cd**	**Sn**	**Sb**	**Pb**
A031	0.04	0.001	0.03	0.01	0.58	0.0004	0.0032	< DL	< DL	< DL	0.0001	< DL	< DL	< DL	0.0000	< DL	0.0000
A051	0.00	0.001	0.08	0.01	0.65	< DL	0.0113	< DL	0.0007	0.0000	0.0001	0.0000	0.0000	0.0000	0.0000	< DL	< DL
A081	0.10	0.002	< DL	0.19	0.73	< DL	0.0108	< DL	< DL	0.0006	0.0005	0.0000	< DL	< DL	0.0004	< DL	< DL
A91	0.87	0.013	0.03	0.14	4.81	0.0020	0.0128	0.4828	0.1530	0.0004	0.0002	0.0001	0.0004	0.0004	0.0156	0.0078	0.0211
A124	1.67	0.000	0.37	0.00	0.56	0.1457	0.0001	0.0018	0.1208	0.0001	0.0002	0.0001	0.0000	0.0001	0.0039	0.0005	0.0004
A131	0.20	0.001	0.00	0.01	1.54	< DL	0.0019	2.4611	3.5646	0.0009	0.0010	0.0003	0.0000	0.0023	0.0038	0.0012	0.0437
A13	1.40	0.002	0.20	0.03	1.97	0.0014	0.0207	4.5463	0.5995	< DL	0.0026	0.0001	0.0001	0.0003	0.0163	0.0022	1.6258
A134	1.65	0.000	< DL	0.01	2.07	0.0056	0.0793	0.0293	0.3759	< DL	0.0002	0.0016	< DL	0.0001	0.0008	0.0134	0.0011
A136	7.99	0.002	0.01	0.01	0.61	0.0186	0.0015	1.6894	4.2354	0.0010	0.0013	0.0002	0.0001	0.0001	0.0008	0.0226	0.0005

Table S5. Concentrations of metals in the test medium as a result of addition of fire ash from different sources including vegetation, (A31, A51, and A81) structures, (A91, A124, and A131) vehicles (A13, A134, and A136). The Fe content was fixed for all exposures at 50 μM Fe.

**Mean**	**Ti**	**V**	**Cr**	**Mn**	**Fe**	**Co**	**Ni**	**Cu**	**Zn**	**As**	**Se**	**Mo**	**Ag**	**Cd**	**Sn**	**Sb**	**Pb**
A031	2.41	0.06	1.08	1.01	50.0	0.061	0.868	< DL	< DL	< DL	0.004	< DL	< DL	< DL	0.003	< DL	0.002
A051	2.12	0.08	1.19	1.66	50.0	< DL	0.909	< DL	< DL	0.010	0.008	0.000	0.005	0.003	0.002	< DL	< DL
A081	7.24	0.12	< DL	17.65	50.9	< DL	0.912	< DL	< DL	0.024	0.021	0.001	< DL	< DL	0.022	< DL	< DL
A91	19.98	0.08	0.24	1.18	42.9	0.019	0.119	5.565	1.703	0.013	0.008	0.002	0.006	0.015	0.194	0.135	0.159
A124	65.42	0.06	16.99	0.22	35.7	2.477	0.073	0.101	5.160	0.011	0.012	0.005	0.003	0.006	0.027	0.020	0.004
A131	17.38	0.12	0.50	0.62	42.9	< DL	0.143	11.280	141.201	0.042	0.046	0.007	0.005	0.042	0.067	0.115	0.709
A13	102.08	0.07	0.83	2.47	50.0	0.056	0.757	27.391	16.932	< DL	0.029	0.005	0.003	0.005	0.125	0.233	9.724
A134	48.69	0.04	< DL	0.36	41.7	0.147	0.439	0.913	33.373	< DL	0.012	0.014	< DL	0.002	0.038	0.132	0.054
A136	547.35	0.23	0.45	1.05	50.0	1.114	0.260	131.489	319.229	0.088	0.118	0.015	0.006	0.013	0.105	1.397	0.276
**Standard deviation**	**Ti**	**V**	**Cr**	**Mn**	**Fe**	**Co**	**Ni**	**Cu**	**Zn**	**As**	**Se**	**Mo**	**Ag**	**Cd**	**Sn**	**Sb**	**Pb**
A031	0.19	0.00	0.14	0.03	2.9	0.002	0.016	< DL	< DL	< DL	0.000	< DL	< DL	< DL	0.000	< DL	0.000
A051	0.00	0.01	0.39	0.06	3.2	< DL	0.056	< DL	0.003	0.000	0.000	0.000	0.000	0.000	0.000	< DL	< DL
A081	0.48	0.01	< DL	0.94	3.7	< DL	0.054	< DL	< DL	0.003	0.002	0.000	< DL	< DL	0.002	< DL	< DL
A91	4.34	0.06	0.15	0.69	24.1	0.010	0.064	2.414	0.765	0.002	0.001	0.000	0.002	0.002	0.078	0.039	0.106
A124	8.34	0.00	1.86	0.02	2.8	0.729	0.000	0.009	0.604	0.001	0.001	0.001	0.000	0.000	0.020	0.003	0.002
A131	1.00	0.01	0.02	0.07	7.7	< DL	0.009	12.302	17.818	0.004	0.005	0.001	0.000	0.012	0.019	0.006	0.219
A13	6.99	0.01	0.99	0.15	9.8	0.007	0.103	22.731	2.997	< DL	0.013	0.000	0.000	0.001	0.082	0.011	8.129
A134	8.25	0.00	< DL	0.06	10.4	0.028	0.397	0.147	1.881	< DL	0.001	0.008	< DL	0.000	0.004	0.067	0.005
A136	39.98	0.01	0.04	0.06	3.0	0.093	0.008	8.449	21.182	0.005	0.006	0.001	0.001	0.001	0.004	0.113	0.003

DL: detection limit.

Table S6. Pearson and spearman correlation analysis between metal concentrations and *Thalassiosira weissflogii* growth rate (GR).

**Correlation**	**Pearson test**	***p-value***	**Spearman test**	***p-value***
Ti vs. GR	-0.519	0.027	-0.624	0.007
V vs. GR	-0.509	0.031	-0.426	0.079
Cr vs. GR	-0.015	0.952	0.052	0.838
Mn vs. GR	0.208	0.407	0.079	0.754
Fe vs. GR	-0.162	0.521	0.005	0.987
Co vs. GR	-0.243	0.331	-0.301	0.225
Ni vs. GR	0.368	0.133	0.385	0.116
Cu vs. GR	-0.531	0.023	-0.723	0.001
Zn vs. GR	-0.728	0.001	-0.75	0.0003
As vs. GR	-0.668	0.002	-0.551	0.018
Se vs. GR	-0.652	0.003	-0.649	0.004
Mo vs. GR	-0.653	0.003	-0.692	0.001
Ag vs. GR	-0.486	0.041	-0.457	0.056
Cd vs. GR	-0.698	0.001	-0.663	0.003
Sn vs. GR	-0.377	0.123	-0.682	0.002
Sb vs. GR	-0.557	0.016	-0.708	0.001
Pb vs. GR	0.086	0.734	-0.598	0.009
